# Live Attenuated Influenza Vaccine contains Substantial and Unexpected Amounts of Defective Viral Genomic RNA

**DOI:** 10.3390/v9100269

**Published:** 2017-09-21

**Authors:** Philip S. Gould, Andrew J. Easton, Nigel J. Dimmock

**Affiliations:** 1 Faculty of Health and Life Sciences, Coventry University, Science and Health Building, 20 Whitefriars Street Coventry CV1 2DS, UK; Phillip.gould@coventry.ac.uk; 2 School of Life Sciences, University of Warwick, Coventry CV4 7AL, UK

**Keywords:** influenza A, influenza B, live attenuated vaccine, defective interfering RNA

## Abstract

The live attenuated influenza vaccine FluMist^®^ was withdrawn in the USA by the Centers for Disease Control and Prevention after its failure to provide adequate protective immunity during 2013–2016. The vaccine uses attenuated core type A and type B viruses, reconfigured each year to express the two major surface antigens of the currently circulating viruses. Here Fluenz™ Tetra, the European version of this vaccine, was examined directly for defective-interfering (DI) viral RNAs. DI RNAs are deleted versions of the infectious virus genome, and have powerful biological properties including attenuation of infection, reduction of infectious virus yield, and stimulation of some immune responses. Reverse transcription polymerase chain reaction followed by cloning and sequencing showed that Fluenz™ vaccine contains unexpected and substantial amounts of DI RNA arising from both its influenza A and influenza B components, with 87 different DI RNA sequences identified. Flu A DI RNAs from segment 3 replaced the majority of the genomic full-length segment 3, thus compromising its infectivity. DI RNAs arise during vaccine production and non-infectious DI virus replaces infectious virus pro rata so that fewer doses of the vaccine can be made. Instead the vaccine carries a large amount of non-infectious but biologically active DI virus. The presence of DI RNAs could significantly reduce the multiplication in the respiratory tract of the vaccine leading to reduced immunizing efficacy and could also stimulate the host antiviral responses, further depressing vaccine multiplication. The role of DI viruses in the performance of this and other vaccines requires further investigation.

## 1. Introduction

Influenza occurs mainly as a seasonal winter-time respiratory infection in Northern and Southern hemispheres, with both influenza A and B viruses being responsible. The many subtypes of influenza A viruses are determined by the major surface antigens of influenza viruses, the haemagglutinin (HA) and neuraminidase (NA) proteins. A and B viruses both undergo antigenic drift, a continuous mutation of the genes encoding the HA and NA proteins that results in antigenic changes which, over a period of approximately 4 years, render previously acquired immunity ineffective [1]. In addition, influenza A viruses undergo antigenic shift, a property that stems from the ability of two or more subtypes to exchange genomic segments through RNA reassortment. This results in the formation of novel chimeric viruses derived from human and/or non-human strains that can cause pandemics of influenza. Influenza B viruses undergo antigenic drift but not shift, and drift has given rise to two distinct antigenic lineages that currently co-circulate.

The drain on national economies from annual influenza outbreaks is significant and has been estimated to cost the USA at least $90 billion per year [2]. Vaccines are the main public health measure used to prevent influenza caused by A and B viruses, with the antivirals oseltamivir and zanamivir employed to prevent and treat infections. The first influenza vaccine established was an inactivated virus preparation. It now contains separated and purified HA and NA proteins of currently circulating viruses, and is injected intramuscularly. In 2003 an intranasally administered live attenuated vaccine was introduced under the name FluMist^®^ predominantly with the aim of protecting children [3]. This was upgraded in 2013 to a tetravalent preparation containing 2 recombinant influenza A and 2 recombinant B viruses (Table 1). At its peak more than 14 million doses were manufactured and distributed worldwide each year. FluMist^®^ is licensed in the USA for those aged 2 to 49 years. In Europe, the same vaccine is marketed as Fluenz™ Tetra and is used to prevent influenza in those aged 2 to 18 years. The Centers for Disease Control and Prevention (CDC) Advisory Committee on Immunization Practices (ACIP) determined that FluMist^®^, the live attenuated influenza vaccine (LAIV), should not be used during the 2016–2017 flu season in the USA based on data showing poor or relatively lower effectiveness of LAIV from 2013 to 2016 [4].The European version of the vaccine is Fluenz™ Tetra and this is being monitored more intensively than other medicines by the European Medicines Agency (EMA) [5].

Fluenz™ contains the genomic RNAs encoding the HA and NA proteins of selected circulating A or B strains and 6 other genomic RNAs from an attenuated influenza A/Ann Arbor/6/1960 or influenza B/Ann Arbor/1/1966 master donor strain, respectively. The master donor strains were made by adaption to growth in cell culture at subphysiological temperatures, and both have the phenotypic characteristics of cold adaption (*ca*) (efficient growth at 25 °C), temperature sensitivity (*ts*) in cell culture of influenza A at 39 °C and influenza B at 37 °C, and attenuation (*att*) or restricted growth in the lower respiratory tract of ferrets [6]. There are multiple nucleotide substitutions spread over most of the 6 RNAs, protecting against reversion to wild-type. Each of the four vaccine strains is grown in embryonated chicken’s eggs and then diluted to the desired infectious concentration to form the tetravalent vaccine.

**Figure 1:**
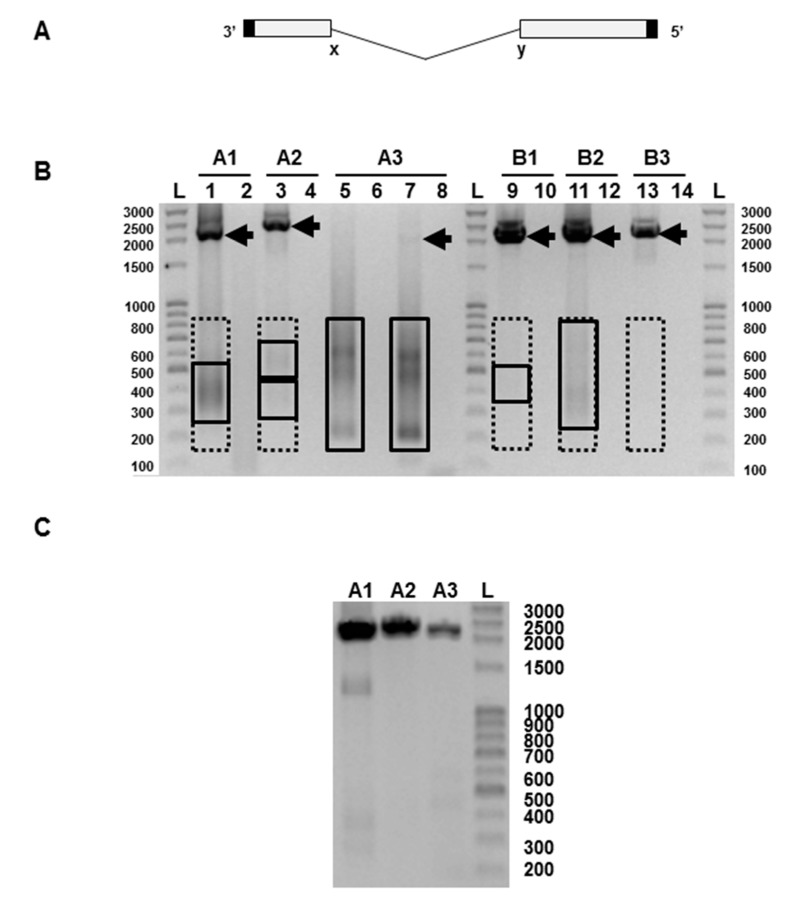
(**A**) Diagram of the general genetic organisation of influenza virus defective-interfering (DI) RNAs. The central deletion between positions labelled x and y is highly variable in length but the 5′ and 3′ termini (solid) are retained. (**B**)Representative analysis of the products of reverse transcriptase-polymerase chain reaction (RT-PCR) using primers specific for each of segments 1, 2, and 3 of the influenza A and influenza B strains present in the Fluenz™ Tetra vaccine (batch CH2020). Products representing full-length influenza segments are indicated by arrows; boxed areas indicate the regions excised for gel extraction for putative DI RNAs. Influenza A segment 1 (lanes 1–2), segment 2 (lanes 3–4), segment 3 (lanes 5–8). Influenza B segment 1 (lanes 9–10), segment 2 (lanes 11–12), segment 3: (lanes 13–14). Products were amplified for 30 cycles except for lanes 7–8 which required 35 cycles to visualise a full-length segment 3 RNA. Odd numbers show the products of a reaction mix containing reverse transcriptase (RT) while even numbers lack reverse transcriptase. (**C**) Reverse transcriptase-PCR of the vaccine passaged once at limiting dilution in embryonated hen’s eggs showing full-length segments A1–3. The expected sizes of the PCR fragments derived from the full-length RNAs are: A1 2239 nts, A2 2318 nts, A3 2184 nts, B1 2235 nts, B2 2306 nts, and B3 2235 nts. L, indicates a ladder of markers with the size indicated in nucleotides.

Non-infectious defective interfering (DI) influenza viruses commonly occur in preparations grown in vivo [7], in vitro [8,9], and in natural human infections [10]. The rate of generation and accumulation of DI virus depends on the virus strain, the host cell, and the manner of propagation, and they become more abundant when the virus is propagated at a high multiplicity of infection [7,11]. DI viruses are *defective* through having a major deletion in the genome, and *interfering* as the DI genome has the ability to inhibit the production of infectious virus [12]. Influenza A DI RNAs all have a major deletion (around 80%) from the central region of the cognate full-length RNA segment and typically comprise 400–500 nucleotides [13,14,15]. There are no published data for influenza B DI RNAs. Importantly, the positions of the two major break points within the genomic RNAs are highly variable. Thus, there is a heterogeneous population of RNAs that vary in size, sequence, and segment of origin. However, the termini of the genomic RNA are always conserved (Figure 1A). Any genome segment can give rise to DI RNAs but most arise from RNAs 1, 2, and 3 which encode the 3 proteins that make up the virion RNA-dependent RNA polymerase (PB2, PB1, and PA) [16]. The DI RNA has a replication rate advantage over its cognate full-length segment and is preferentially packaged [17]. Thus a virus particle containing a DI RNA is by definition non-infectious, and a preparation having a high DI virus content will have a high particle: infectivity ratio. One cloned DI RNA derived from genome segment 1 has been shown to specifically interfere with RNA synthesis from segments 1, 2, and 3, further compromising the production of infectious virus [18]. If DI virus is added to infectious virus in vitro, infection can be inhibited to the extent of completely abrogating cytopathology, while in vivo DI virus can reduce an otherwise fatal disease to a subclinical infection [17]. Here we have directly analysed Fluenz™ Tetra influenza vaccine and find that it contains substantial amounts of influenza DI RNAs.

As all influenza A DI RNAs retain the terminal sequences of the full-length RNA but lack most of its internal sequence, our strategy was to use primers designed to anneal at the termini of the genome segments and RT-PCR to amplify the relevant viral RNAs. We examined full-length segment 1, 2, or 3 RNAs and subgenomic RNAs derived therefrom. To discriminate between products originating from each of the three segments it was necessary to design primers that annealed to unique sequences adjacent to the conserved termini. Although influenza B DI viruses are well known, no influenza B DI RNA sequence has been reported.

## 2. Materials and Methods

### 2.1. Ethics Statement

Experiments involving animals were approved in June 2014 by the University of Warwick Animal Welfare and Ethical Review Board as required by the Animals (Scientific Procedures) Act (1986) governing animal experimentation in the UK.

### 2.2. RNA Isolation

RNA was isolated from Fluenz™ Tetra from the 2014–2015 vaccine season (batches CH2020 and CH2065) using Trizol LS (Invitrogen, Paisley, Scotland, UK) according to the manufacturers’ recommendations.

### 2.3. Reverse Transcription-polymerase Chain Reaction (RT-PCR)

cDNA was made using reverse transcriptase Superscript III (Invitrogen, Paisley, Scotland, UK) with 60 minutes extension at 55 °C and the post extraction RNaseH option, using general segment-specific primers for type A (NNNAGCAAAAGCAGG) and type B (AGCAGAAGCGGWGCGTTT). Each segment was amplified with Pfu DNA polymerase (Promega, Madison, Wisconsin, USA) for 30 seconds at 94 °C and then 30 cycles of 30 seconds at 94 °C, 30 seconds at 55 °C, 5 minutes at 68 °C, and a final extension for 10 minutes at 72 °C. The primers for the A/Ann Arbor/6/1960 (H2N2) master donor strain were: RNA1 forward primer (A1For) (^60^GTCGCAGTCCCGCACTCGCGAG) and RNA1 reverse primer (A1Rev) (^2298^GGCCATCCGAATTCTTTTGG); A2For (^12^GCAAACCATTTGAATGGATG) and A2Rev (^2329^CATTTTTTCATGAAGGACAAG); A3For (^28^GAAGACTTTGTGCGACAATGC) and A3Rev (^2211^GGACAGTACGGATAACAAATAG). The number of the 5′ terminal nucleotide refers to the nucleotide position in the genome segment to which the primer anneals. The primers for the B/Ann Arbor/1/1966 master donor strain were: B1For (^32^ATCCTTATTTTCTCTTCATAGATG), B1rev (^2266^TCTCACCAAGGTGAGCCATTGC); B2For (^33^GCCAAAATTGAATTGTTAAAACAAC), B2Rev (^2338^TATTAGCTCAAGGCCCACCC); B3For (^32^GGATACTTTTATTACAAGAAAC), and B3Rev (^2266^GATGTTTAGATACATAATGAAC).

### 2.4. Growth of Virus in Embryonated Chicken’s Eggs 

Vaccine was inoculated at limiting dilution into the allantoic cavity of 10-day old embryonated eggs which were incubated for 48 h at 31 °C. Allantoic fluid was tested for virus by haemagglutination assay and virus-positive material from the highest dilution inoculated was tested by RT-PCR.

### 2.5. Haemagglutination Assay

The 50% haemagglutination titre of Fluenz™ Tetra was determined in a standard assay. Fluenz™ Tetra was diluted in 2-fold serial steps in PBS in a 96-well plate to give a final volume of 100 µL per well. A 30 µL aliquot of a 1% suspension of chicken red blood cells was added to each well, mixed and left for 45 min at ambient temperature. An aliquot of influenza A/Puerto Rico/8/34 virus was used as a positive control. 

## 3. Results

PCR products were analysed by gel electrophoresis and the results for vaccine batch CH2020 are shown in Figure 1B. Vaccine batch CH2065 gave very similar data (not shown). The full-length RNAs, with the exception of influenza A segment 3, were clearly visible. Segment A3 could be seen as a faint band when the PCR was continued for an additional 5 cycles. To verify that this was a genuine finding and not a failure of the RT-PCR, we passaged the vaccine once at limiting dilution in embryonated chicken’s eggs, a procedure known to minimise the amount of DI RNA present [7]. PCR of the allantoic fluid from one such egg showed PCR products derived from the full length segments A1, A2, and A3 (Figure 1C), and confirms the validity of the PCRs in Figure 1B. In Figure 1B PCR products representing putative DI RNAs in the 200–800 nt size range can be seen on some tracks as smears or fuzzy bands, as expected from the variable nature of the deletion and the heterogeneity of the resulting RNAs. These were most intense in the influenza A RNA segment 3 track where the PCR product of the full-length A3 RNA was very faint. Equivalent PCR products were also seen in the egg-passaged material (Figure 1C), though at much lower levels, as expected [7], and these were not analysed further. Bands representing PCR products amplified directly from the Fluenz™ Tetra vaccine batch CH2020 and derived from full-length RNAs and putative DI RNAs were extracted from the gel, cloned using the Zero Blunt PCR cloning kit (Invitrogen, Paisley, Scotland, UK), and sequenced. This confirmed that influenza A and B segment 1–3 RNAs were derived from the relevant master donor strain [19,20]. Amplified DNA derived from putative DI RNAs obtained from influenza A segment 2, and influenza B segments 1 and 3 were weak or not visible, but gel extraction of the anticipated region (200–800 nts) was carried out and DI RNA sequences were found in all samples (Figure 1B, Table 1). Sequenced RNAs were aligned with RNAs 1–3 of the parental strains (A/Ann Arbor/6/1960 and B/Ann Arbor/1/1966) [19,20] using DNASTAR software. Data show that subgenomic RNAs have the terminal sequences and extensive central deletion typical of an influenza DI RNA (Table 1). Eighty-seven unique DI RNA sequences were identified. Most had a single central deletion but a majority of A2 DI RNAs (61.5%) had a more complex deletion pattern. Only one DI RNA sequence was isolated on more than one occasion (Table 1). The flu A and B DI RNA 5′ and 3′ end breakpoints were highly variable in terms of both position and nucleotide sequences with no consistent pattern seen with either virus. No point mutations that would arise from misincorporation were detected.

The infectiveness of an influenza virus preparation is determined by comparing the infectivity itself (infectious units or IU) with the total number of particles present. Infectious and non-infectious virus particles agglutinate red blood cells equally, so the total particle count can be conveniently measured by haemagglutination (in haemagglutinating units or HAU) with chicken red blood cells. Infectiveness is expressed as an IU:HAU ratio, with the most infectious preparations having a ratio of 10^6^. The haemagglutinin titre of Fluenz™ was determined as 2 × 10^3^ HAU/0.2 mL. The infectivity of Fluenz™ is 10^7^ infectious units/0.2 mL for each of the four virus strains (manufacturer’s specification), giving an IU:HAU ratio of 5 × 10^3^ for at least one of the four components of the vaccine. Such a low ratio, over 99% below the maximum value, is consistent with the presence of substantial amounts of DI RNA and, by implication, of DI virus. This means that the vaccine contains a vast excess of non-infectious virus.

## 4. Discussion

The presence of DI RNA sequences in commercial live attenuated influenza vaccine has not previously been reported, although there was speculation in a preliminary report [21]. Earlier work passaged vaccines in cells to enhance the presence of DI viruses but DI viruses could have been generated de novo during these passages [22,23,24]. One report suggested that a 250 to 2000-fold concentrated pre-vaccine preparation of measles virus culture fluid contained subgenomic RNAs, but these were uncharacteristically large for DI RNAs, and were not analysed for sequence or interference [25]. A single DI RNA sequence was isolated from an attenuated Chinese measles vaccine [26]. Deep sequencing of vaccine preparations of poliovirus (types 1, 2, and 3), measles virus, mumps virus, and rotavirus (Rotateq^®^), yellow fever virus, and human herpes 3 (varicella zoster) virus showed no evidence of over-representation of terminal sequences that would be expected with DI virus genomes [27]. The genome coverage of rubella virus (Meruvax^®^ II and MMR^®^ II) and rotavirus (Rotarix^®^) was not sufficient to allow conclusions to be drawn about the possible presence of DI virus genomes.

Generally, influenza viruses are passaged at low multiplicity of infection to avoid accumulation of DI RNAs and the reduction in virus yield that ensues [7]. However, production details for Fluenz™ have not been published. Different influenza strains vary greatly in their ability to accumulate DI virus [28,29], while mutations in PA [30] or NS2 [31,32] increased the accumulation of influenza DI RNAs. It is not known if the original viruses used to derive the Fluenz™ master donor strains are particularly prone to accumulate DI RNAs, whether this is influenced by the mutations acquired by the two master donor strains during cold-adaptation, or whether the DI viruses arise as a result of particular steps in the manufacturing process which may promote their formation.

The relative abundances of the full-length genome segments and the DI RNAs derived from them cannot be accurately determined using a PCR-based approach as primers based on the termini of the genome will amplify RNA molecules of significantly different length with different efficiencies. Further, a quantitative RT-PCR assay for heterogeneous DI RNAs that does not also amplify the cognate full-length genome segment requires a DI RNA-specific primer to cover the novel junction point generated by the deletion event, and this is not possible for a population of DI RNAs of hugely variable sequence. We have relied on qualitative differences in amplification of full-length genome or DI RNAs, but these can be significant, as demonstrated by the low levels of full-length segment 3 of influenza A virus.

An influenza virus particle packages one of each of the 8 RNA segments but does not appear to distinguish between a full-length RNA and its cognate DI RNA, both possessing the same terminal packaging signals. Any virion packaging a DI RNA lacks the coding capacity of that full-length segment and is, by definition, non-infectious [17], and any DI RNA in a virus preparation reduces its infectivity pro rata. The severe reduction of full-length segment 3 seen in Figure 1B is indicative of a likely significant loss of infectivity, and is fully consistent with the low infectivity:HAU ratio found.

A DI RNA acts primarily to reduce the replication of a homologous virus, but can also attenuate infection through its interfering properties [17]. Either could lower the efficacy of Fluenz™/FluMist^®^ through reducing the virus load produced in the upper respiratory tract and hence its immunizing ability. Some DI RNAs are immunostimulatory and activate innate immune responses which render them capable of stimulating T cell-mediated immunity. It has been previously argued that DI viruses may act as a natural adjuvant [33,34,35]. Thus, putative DI viruses found in Fluenz™/FluMist^®^ might be a positive benefit to the live influenza vaccine, if somewhat inadvertent and variable. This inconsistency might also contribute to the varied efficacy recently encountered with FluMist^®^ which resulted in the recommendation that live attenuated influenza vaccine (LAIV) should not be used during the 2016–2017 flu season [4].

In conclusion, we have shown that Fluenz™ Tetra preparations contain substantial amounts of putative DI RNAs. The presence of DI RNAs in preparations given to recipients without reports of adverse effects suggests that the DI RNAs in Fluenz™/FluMist^®^ do not present a health problem. However, it would be of considerable public interest to establish if DI RNAs contribute negatively or positively to the ability of Fluenz™/FluMist^®^ to establish protective immunity, particularly in paediatric flu prevention and transmission reduction.

## Figures and Tables

**Table 1:** Summary of DI RNA sequences isolated directly from Fluenz™ Tetra vaccine.

Virus	Segment	Full-Length Segment (nts) ^a^	Total Number of DI RNAs Sequenced	Complex DI RNAs ^b^	Range in Position of First Breakpoint ^c^	Range in Position of Last Breakpoint	Range in Size of DI RNAs (nts)	Median DI RNA (nts)	Mean DI RNA (nts)
A/Ann Arbor/6/60	1	2341	24 (23) ^d^	3/23	102–264	1980–2154	311–587	413	438.3
	2	2341	13	8/13	54–468	1876–2259	206–786	379	454.6
	3	2233	17	5/17	82–319	1829–2257	257–713	447	430.6
B/Ann Arbor/1/66	1	2369	12	1/12	90–511	1730–2218	361–809	537.5	538.6
	2	2396	11	0/11	136–429	1936–2203	387–786	543	573.2
	3	2308	11	0/11	73–326	1954–2139	496–658	540	546.4

The positions of the breakpoints were determined by comparison with the nucleotide sequences of the influenza A/Ann Arbor/6/60 (H2N2) cold adapted strain genome segment 1 (GenBank accession number M23970.1), segment 2 (GenBank accession number M23972.1), and segment 3 (GenBank accession number M23974.1) and influenza B/Ann Arbor/1/66 segment 1 (GenBank accession number M20169.1), segment 2 (GenBank accession number M20163.1), and segment 3 (GenBank accession number M20171.1); ^a^ nts, nucleotides; ^b^ Most DI RNAs have a single central deletion with two breakpoints; others indicated here have one or more additional breakpoints (not shown) but all retain the terminal sequences of the full-length segment; ^c^ in nucleotides from 3′–5′ of virion sense RNA; ^d^ One DI RNA sequence (single central deletion) was found twice in the A1-derived DI RNAs and was discounted for further calculation.
